# AC Current‐Driven Magnetization Switching and Nonlinear Hall Rectification in a Magnetic Topological Insulator

**DOI:** 10.1002/adma.202506210

**Published:** 2025-10-14

**Authors:** Yuto Kiyonaga, Masataka Mogi, Ryutaro Yoshimi, Yukako Fujishiro, Yuri Suzuki, Max T. Birch, Atsushi Tsukazaki, Minoru Kawamura, Masashi Kawasaki, Yoshinori Tokura

**Affiliations:** ^1^ Department of Applied Physics and Quantum‐Phase Electronics Center (QPEC) The University of Tokyo Tokyo 113‐8656 Japan; ^2^ RIKEN Center for Emergent Matter Science (CEMS) RIKEN Wako 351‐0198 Japan; ^3^ Department of Advanced Materials Science The University of Tokyo Kashiwa 277‐8561 Japan; ^4^ RIKEN Cluster for Pioneering Research (CPR) RIKEN Wako 351‐0198 Japan; ^5^ Tokyo College The University of Tokyo Tokyo 113‐8656 Japan

**Keywords:** frequency mixing, magnetization switching, nonlinear Hall effect, spin–orbit torque, topological insulator

## Abstract

Spin–orbit torque arising from the spin–orbit‐coupled surface states of topological insulators enables current‐induced control of magnetization with high efficiency. Here, alternating‐current (AC) driven magnetization reversal is demonstrated in a semi‐magnetic topological insulator (Cr,Bi,Sb)_2_Te_3_/(Bi,Sb)_2_Te_3_, facilitated by a low threshold current density of 1.5 × 10^9^ A m^−2^. Time‐domain Hall voltage measurements using an oscilloscope reveal a strongly nonlinear and rectified Hall response during the magnetization reversal process. Fourier analysis of the time‐varying Hall voltage identifies higher‐harmonic signals and a rectified direct‐current (DC) component, highlighting the complex interplay among the applied current, external magnetic field, and magnetization dynamics. Furthermore, a hysteretic behavior in the current‐voltage characteristics gives rise to frequency mixing under dual‐frequency excitation. This effect, distinct from conventional polynomial‐based nonlinearities, allows for selective extraction of specific frequency components. The results demonstrate that AC excitation can not only switch magnetization efficiently but also induce tunable nonlinear responses, offering a new pathway for multifunctional spintronic devices with potential applications in energy‐efficient memory, signal processing, and frequency conversion.

## Graphical abstract

This study reports alternating‐current (AC) driven magnetization reversal in a semi‐magnetic topological insulator (Cr,Bi,Sb)_2_Te_3_/(Bi,Sb)_2_Te_3_, with low current density. Time‐domain Hall voltage measurements reveal a nonlinear and rectified Hall effect, along with higher‐harmonic responses and hysteresis. These findings, including asymmetric frequency mixing, offer potential for spintronic applications like signal processing and frequency conversion.

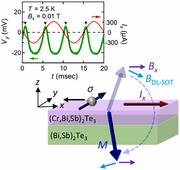

## 1 Introduction

The interplay of electron spin and charge transport underlies a wide range of spintronic phenomena and applications, including, for example, current‐induced magnetization reversal in ferromagnets mediated by spin‐transfer and spin–orbit torques.^[^ ^1^, ^2^, ^3^ ^]^ In addition to driving magnetization dynamics, localized spins significantly influence the charge transport properties, leading to nonreciprocal conduction,^[^ ^4^, ^5^ ^]^ spin‐motive forces,^[^ ^6^, ^7^ ^]^ and emergent electric fields.^[^ ^8^, ^9^, ^10^ ^]^ These effects open possibilities for advanced functionalities, such as diode operation, direct‐current (DC) rectification, frequency conversion, and novel inductive elements.^[^ ^10^, ^11^ ^]^

Nonlinear Hall effects arising from such spin‐charge coupling also offer a means to detect current‐driven magnetization dynamics.^[^ ^12^, ^13^ ^]^ In particular, second harmonic Hall voltages generated by continuous alternating‐current (AC) excitation are commonly employed to probe magnetization oscillations.^[^ ^13^ ^]^ However, driving magnetization dynamics typically requires large current densities on the order of 10^10^ to 10^11^ A m^−2^, often applied as short pulses to mitigate Joule heating,^[^ ^1^ ^]^ posing multiple challenges originating from extrinsic thermal effects, including parasitic Nernst effects,^[^ ^14^ ^]^ spin‐Seebeck effects,^[^ ^15^ ^]^ and enhanced magnon scattering.^[^ ^4^, ^5^ ^]^ These parasitic effects often mask the intrinsic nonlinear signals that stem directly from magnetization dynamics. To date, a nonlinear Hall effect directly associated with magnetization reversal driven by spin‐transfer or spin–orbit torques remains largely unexplored.

Time‐domain measurements of the nonlinear Hall effect provide an effective method to investigate magnetization dynamics. Such measurements have been employed to study ultrafast magnetization switching,^[^ ^16^, ^17^ ^]^ magnetic domain wall motion induced by pulse current,^[^ ^18^ ^]^ and inertial skyrmion dynamics under AC currents.^[^ ^19^ ^]^ In our study, recording the current–Hall‐voltage waveform enables simultaneous access to higher harmonics (including DC rectification), hysteretic switching trajectories, and the timing of transitions, making time‐domain measurements well suited to AC‐driven nonlinear Hall responses. In addition, simultaneous monitoring of the longitudinal resistance as a thermometer enables evaluation of thermal effects and thereby separation from intrinsic nonlinear signals from parasitic heating.

Topological insulators (TIs) serve as a unique platform to explore these effects, as they host insulating bulk states and conducting surface states with strong spin–orbit coupling, where electron spin is locked perpendicularly to its momentum.^[^ ^20^ ^]^ This property facilitates efficient spin‐charge conversion via spin–orbit torques (SOTs).^[^ ^21^ ^]^ Indeed, in heterostructures composed of TIs and various ferromagnets, current‐driven magnetization switching has been demonstrated at low current densities on the order of 10^9^ to 10^10^ A m^−2^.^[^ ^13^, ^22^, ^23^ ^]^ Furthermore, when magnetism is introduced into the topological surface state, a magnetization gap is induced, leading to a large anomalous Hall conductivity due to Berry curvature.^[^ ^24^, ^25^ ^]^ Nonetheless, as in conventional ferromagnets, nonlinear transport arising from magnetization dynamics can still be masked by substantial magnon scattering under low current excitation.^[^ ^4^, ^5^ ^]^

In this study, we demonstrate AC current‐induced magnetization dynamics and its associated nonlinear Hall effect in a semi‐magnetic TI (Cr,Bi,Sb)_2_Te_3_/(Bi,Sb)_2_Te_3_ heterostructure thin film. We directly observe magnetization reversal in response to AC current drive by measuring the Hall voltage in time domain using an oscilloscope. The measured Hall voltage exhibits strong current nonlinearity and higher‐harmonic signals, as well as a rectified DC offset. A systematic analysis of the current‐Hall voltage characteristics, combined with Fourier transforms, reveals notable nonlinear and hysteretic responses in the Hall voltage, shedding light on the interplay between current, external magnetic field, and magnetization dynamics. We also identify an asymmetric frequency‐mixing phenomenon originating from the magnetization switching hysteresis, which contrasts with conventional polynomial‐type nonlinearities.

## 2 AC Current‐Induced Magnetization Switching

**Figure 1:**
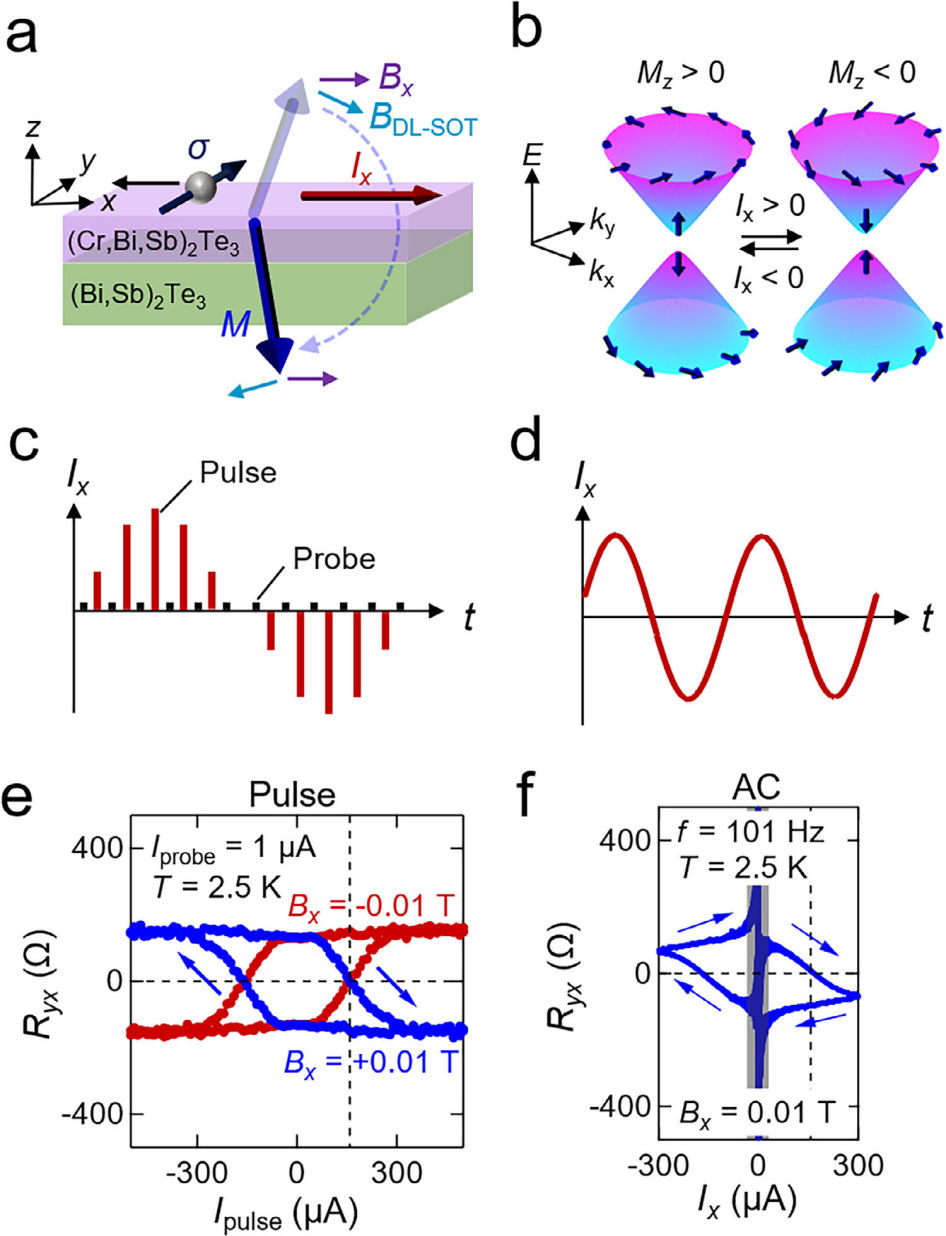
Magnetization switching by pulse current and alternating‐current (AC). a) Schematic illustration of current‐induced magnetization switching. The purple and light blue arrows represent in‐plane magnetic field *B_x_* and the effective magnetic field of damping‐like spin–orbit torque *B*_DL − SOT_, respectively. σ denotes the spin‐polarization of conduction electron. b) Spin‐polarization of gapped Dirac surface states (blue arrows). c,d) Input waveform of pulse and probe current measurement c and alternating current measurement d. e,f) Hall resistance versus applied current for e) pulse and probe and for f) AC. For e) the pulse case, the Hall resistance change is measured by the probe current of 1 µA under the magnetic field *B_x_* of 0.01 T (blue) and − 0.01 T (red) applied parallel to the current direction. The transverse axis shows the pulse current value which varies from − 500 to 500 µA and then from 500 to − 500 µA. For f) the AC case, the Hall resistance is measured by current *I_x_* (*t*) = *I*_peak_ sin (2π*ft*) (*I*_peak_ =  300 µA,  *f*  =  101 Hz) at each time *t* under *B_x_*  =  0.01 T. The black shaded area indicates that the measured value of *R_yx_* diverges near *I_x_* =  0 because it results in an indeterminate form (0/0). The shown data are antisymmetrized with respect to the magnetic field. The vertical dotted lines represent the threshold current where the Hall resistance changes its sign, and the blue arrows represent the direction of the flow of time.

For current‐induced perpendicular magnetization switching in TIs, the damping‐like SOT $\vec{\sigma }\times \left(\vec{\sigma }\times \vec{M}\right)$ plays a central role,^[^ ^3^ ^]^ where σ is the spin polarization of conduction electron and *M* is the localized magnetization. In TIs, the spins are polarized perpendicular to the current when the chemical potential lies in the surface state.^[^ ^24^ ^]^ Therefore, the magnetization with perpendicular magnetic anisotropy is tilted toward the current direction^[^ ^5^, ^13^, ^23^ ^]^ (**Figure** **1**a). The resulting magnetization can be detected by the anomalous Hall effect (AHE) arising from the magnetization gap in the TI surface state (Figure 1b).

We first confirm current‐induced magnetization switching by applying high‐current pulses (up to *I*_pulse_ =   ± 500 µA, or current density of ± 5 × 10^9^ A m^−2^), and under an in‐plane magnetic field *B_x_* =  0.01 T, followed by measuring the Hall voltage at a low sensing current (*I_x_* =  1 µA, or current density of 1 × 10^7^ A m^−2^) (Figure 1c). The Hall voltage *V_y_* is given by *V_y_* = *R_yx_* *I_x_* ∝*M_z_I_x_*, where *M_z_* is the out‐of‐plane component contributing to the AHE. Hence, the sign of the Hall resistance *R_yx_* directly reflects the direction of *M*. By varying the amplitude of the pulse, we observe a clear sign reversal of *R_yx_* at a threshold current of about 150 µA (current density of 1.5 × 10^9^ A m^−2^) (Figure 1e). The switching polarity reverses if we flip either the current direction or the in‐plane field direction, consistent with SOT‐driven perpendicular‐magnetization switching.

Next, instead of applying current pulses, we record the Hall voltage in real time with an oscilloscope (see Methods), while applying an AC current excitation *I_x_* = *I*_peak_ sin(2π*ft*) with a peak current amplitude of *I*_peak_ =  300 µA at a frequency of *f*  =  101 Hz (Figure 1d). We evaluate *R_yx_* from the *R_yx_* = *V_y_* /*I_x_* and plot as a function of *I_x_* in Figure 1f. When 0 µA < *I_x_* < 300 µA, the Hall resistance *R_yx_* gradually decreases and switches polarity above a threshold current (defined where it crosses *R_yx_* =  0) of about 150 µA. Once the polarity is switched, it remains until it is switched back at around *I_x_* =   − 150 µA. This is consistent with the case of pulse‐current‐induced magnetization reversal discussed above. Simultaneous monitoring of the longitudinal resistance *R_xx_* indicates that the temperature is kept below 30 K during the measurement, which is well below *T*_C_ ≈  50 K and no temperature hysteresis was observed (see Note S4, Supporting Information), confirming that Joule heating is not the primary origin of the observed magnetization reversal.

## 3 Nonlinear Hall Voltage and Hysteretic Behavior

**Figure 2:**
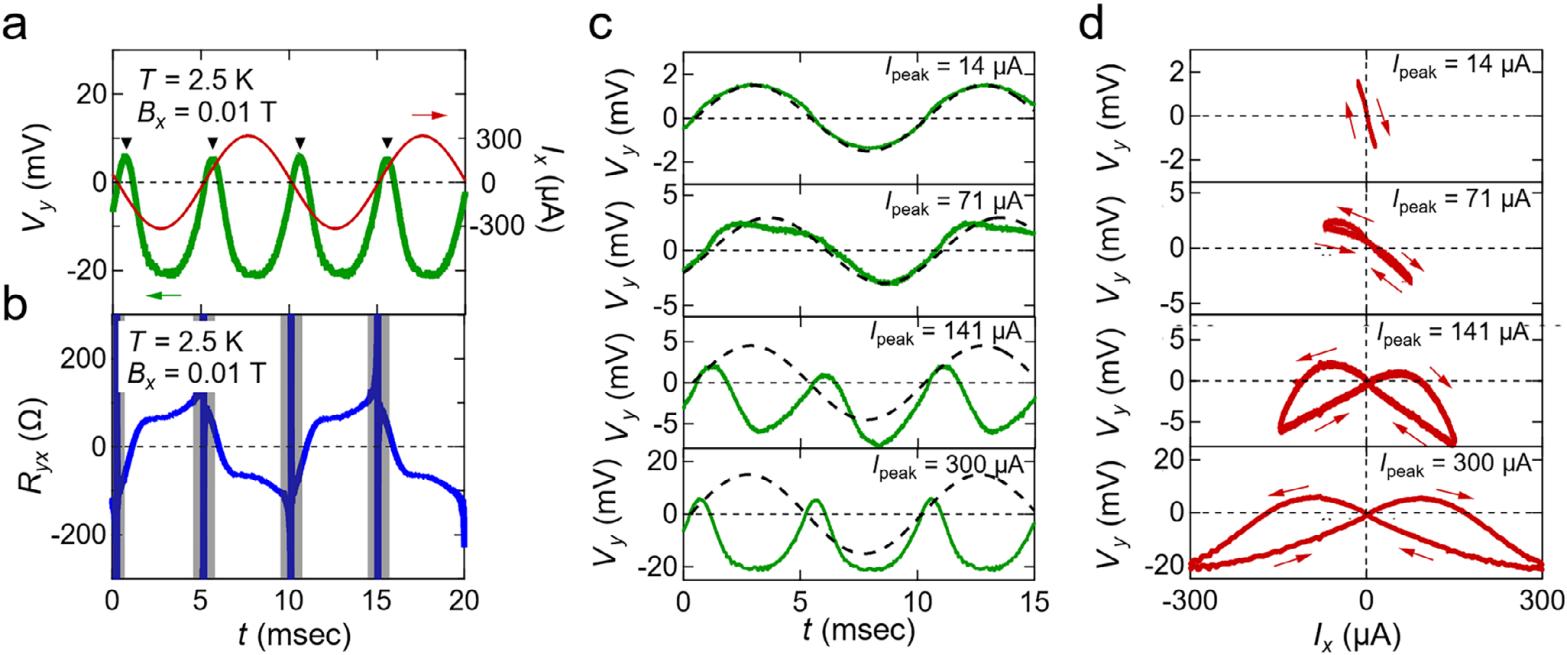
Time domain measurements of magnetization switching. a) Waveforms of input AC current (red, *f*  =  101 Hz) and output Hall voltage (green) measured under in‐plane magnetic field *B_x_* =  0.01 T and temperature *T*  =  2.5 K. Black triangles point to positive spikes of Hall voltage. b) Hall resistance *R_yx_* (blue) calculated from current and Hall voltage shown in (a). Black shaded areas indicate that the value of *R_yx_* diverges around the time period around *I_x_* =  0 because it results in an indeterminate form (0/0). c) Waveforms of the Hall voltage (green) for AC current with amplitudes of 14, 71, 141, and 300 µA. The sinusoidal dotted curve (black) is a guide to the eye which indicates the phase of the AC current. d) Hall voltage versus current in the case of applying AC current whose amplitudes are 14, 71, 141, and 300 µA. The red arrows represent the direction of the flow of time.

**Figure** **2**a shows the time‐dependent evolution of the Hall voltage *V_y_* (t). Since the Hall resistance *R_yx_* becomes negative at *I_x_* = *I*_peak_  and positive at *I_x_* =   − *I*_peak_, as shown in Figure 2b, we see that *V_y_* takes predominantly negative values, reflecting the reversed magnetization state when *I_x_* is above the threshold. Meanwhile, small positive spikes (black triangles in Figure 2a) appear, indicating the short period when the magnetization remains unreversed below the threshold current. To clarify the role of the current amplitude, we compare waveforms of the Hall voltage for several peak current values (Figure 2c). At a low peak current value (*I*_peak_ =  14 µA) which is far below the threshold current, Hall voltage shows a nearly sinusoidal time dependence with alternating signs, originating from the out‐of‐plane component of magnetization under *B_x_* ≈ 0.01 T. With increasing *I*_peak_ (from 71 to 300 µA), the waveforms become increasingly distorted, revealing pronounced nonlinearity in the Hall response. Figure 2d further highlights this nonlinearity by plotting the *I_x_* − *V_y_* relationship for each *I*_peak_. At *I*_peak_ =  14 µA, a nearly linear relationship appears, while at higher currents (e.g., 300 µA), the curves develop a butterfly‐shaped hysteresis: the Hall voltages differ for increasing versus decreasing current. This results from the magnetization dynamics and phase delay when the applied AC current exceeds the threshold of magnetization switching, representing continuous magnetization reversal.

**Figure 3:**
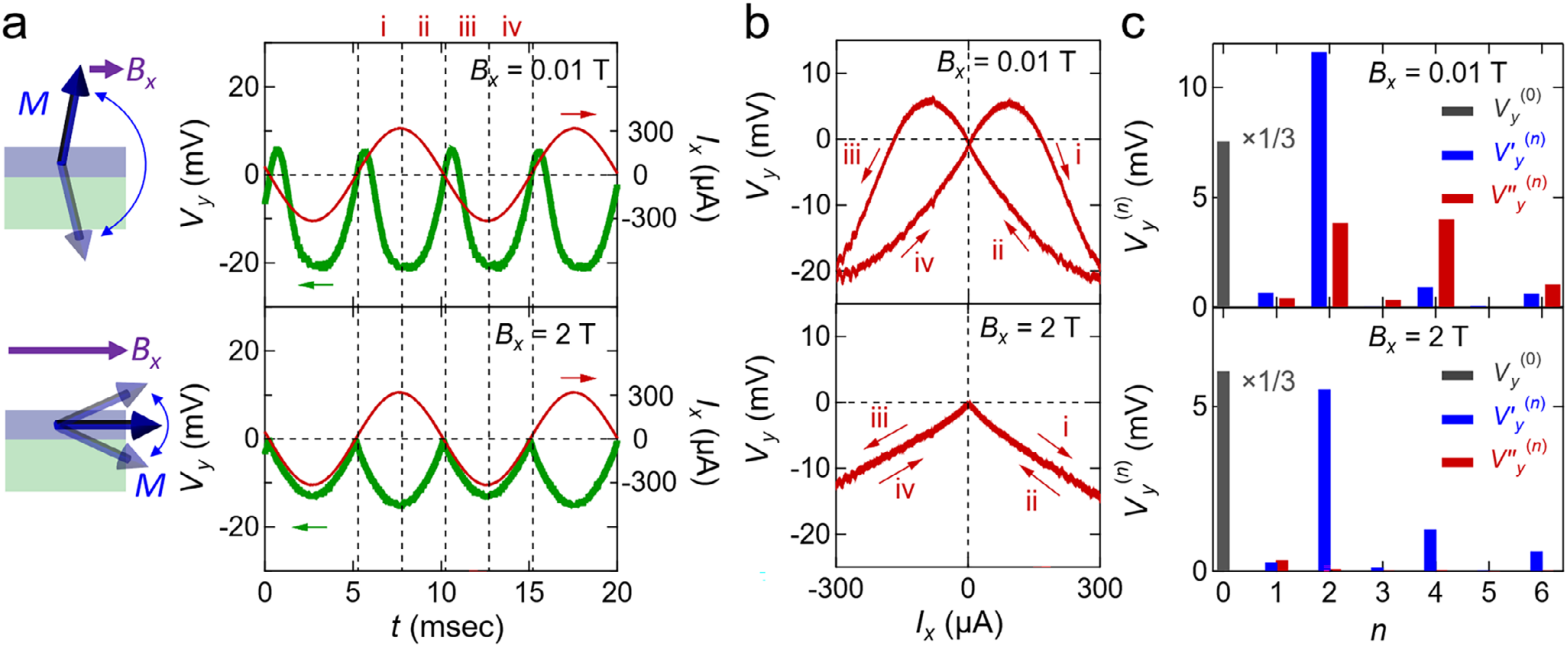
Distinct nonlinear Hall responses under in‐plane magnetic fields. a) Waveform of Hall voltage (green) and current (red) under magnetic field *B_x_*  =  0.01 T (top) and *B_x_*  =  2 T (bottom) and temperature *T*  =  2.5 K. The left illustrations indicate the magnetic field (purple arrows) and the magnetization when applying no current to the sample (dark blue arrows) and the magnetization oscillation (light blue arrows) under the AC current excitation. The vertical dotted lines divide one cycle into 4 regions (i: *dI_x_*/*dt* > 0, *I_x_* > 0, ii: *dI_x_*/*dt* < 0, *I_x_* > 0, iii: *dI_x_*/*dt* < 0, *I_x_* < 0, iv: *dI_x_*/*dt* > 0, *I_x_* < 0). b) Hall voltage versus current under magnetic field *B_x_* = 0.01 T (top) and *B_x_* = 2 T (bottom). The symbols i, ii, iii, and iv on the upper graph corresponds to the ones in a. c) Fourier transformation of the Hall voltage under magnetic field *B_x_*  =  0.01 T (top) and *B_x_*  =  2 T (bottom). The blue, red, and gray bars denote the in‐phase components $V_{y}^{\prime \left(n\right)}$, the out‐of‐phase components $V_{y}^{\prime \prime \left(n\right)}$, and the constant component $V_{y}^{\left(0\right)}$, respectively.

The nonlinear Hall response is also strongly affected by the in‐plane magnetic field altering the magnetization orientation. To investigate this, we perform time‐domain measurements of *V_y_* at a larger field (*B_x_* =  2 T). **Figure** **3**a shows the contrastive Hall voltage waveforms at *B_x_* =  0.01 T and 2 T (the former being the same data from Figure 2a). Unlike at 0.01 T, at 2 T, which is strong enough to force the magnetization to lie in‐plane, no positive spikes appear in regions i and iii of Figure 3a, and the signal remains consistently negative. Correspondingly, the *I_x_* − *V_y_* characteristic shows a complete rectifying behavior without a butterfly shape (Figure 3b, bottom panel). A plausible interpretation is that, under the strong in‐plane field, the magnetization never switches. Instead, magnetization acquires a finite *M_z_* component in the − *z* direction for *I_x_* > 0 and in the + *z* direction for *I_x_* < 0, resulting in a rectified Hall response.

To systematically compare these nonlinear signals, we decompose the time‐domain Hall voltage *V_y_* (*t*) via Fourier transforms. In a simple power‐series expansion of *V_y_* in terms of current *I_x_* (*t*)  =  *I*_0_ sin(ω*t*), *V_y_* (*t*) can be expressed as:

(1) $$\begin{array}{cc} V_{y}\left(t\right) & =V_{y}^{\left(0\right)}+V_{y}^{\prime \left(1\right)}\sin\left(\omega t\right)+V_{y}^{\prime \left(2\right)}\cos\left(2\omega t\right)\\ & \;+V_{y}^{\prime \left(3\right)}\sin\left(3\omega t\right)+V_{y}^{\prime \left(4\right)}\cos\left(4\omega t\right)+\cdot \cdot \cdot \end{array}$$

where $V_{y}^{\left(0\right)}$ and $V_{y}^{\prime \left(n\right)}\;\left(n=1,2,3,4,\cdot \cdot \cdot \right)$ are a constant and coefficients, respectively (see Note S4, Supporting Information, for details). Here all the odd‐harmonic terms appear as sine functions, while all the even‐harmonic ones appear as cosine functions in this power series. More generally, a phase delay arising from magnetization pinning is described by adding odd‐harmonic cosine functions and even‐harmonic sine functions as,

(2) $$\begin{array}{cc} V_{y}\left(t\right) & =V_{y}^{\left(0\right)}+V_{y}^{\prime \left(1\right)}\sin\left(\omega t\right)+V_{y}^{\prime \prime \left(1\right)}\cos\left(\omega t\right)+V_{y}^{\prime \left(2\right)}\cos\left(2\omega t\right)\\ & \;+V_{y}^{\prime \prime \left(2\right)}\sin\left(2\omega t\right)+V_{y}^{\prime \left(3\right)}\sin\left(3\omega t\right)+V_{y}^{\prime \prime \left(3\right)}\cos\left(3\omega t\right)\\ & \;+V_{y}^{\prime \left(4\right)}\cos\left(4\omega t\right)+V_{y}^{\prime \prime \left(4\right)}\sin\left(4\omega t\right)+\cdot \cdot \cdot \end{array}$$

where $V_{y}^{\prime \prime \left(n\right)}\;\left(n=1,2,3,4,\cdot \cdot \cdot \right)$ are coefficients of these phase‐shifted terms. Figure 3c shows the Fourier amplitudes at *B_x_* =  0.01 T and 2 T. In this figure, gray, red, and blue bars represent $V_{y}^{\left(0\right)}$, $V_{y}^{\prime \left(n\right)}$, and $V_{y}^{\prime \prime \left(n\right)}$, respectively. Both cases exhibit strong second‐harmonic $V_{y}^{\prime \left(2\right)}$ appear, corresponding to the fact that *V_y_* remains negative for both *I_x_* = *I*_peak_  and *I_x_* =   − *I*_peak_. However, additional phase‐shifted terms represented as $V_{y}^{\prime \prime \left(n\right)}$ appear only at *B_x_* =  0.01 T, reflecting the delayed response due to magnetization pinning. In contrast, at 2 T, the magnetization follows *I_x_* more smoothly, eliminating large phase shifts. The results are also confirmed by lock‐in measurements of second‐harmonic Hall responses, as shown in Figure S8 (Supporting Information). While lock‐in detection is highly effective for extracting the amplitude and phase of a selected harmonic, it is inherently limited to single‐frequency analysis. By contrast, time‐domain measurements provide direct access to the full waveform of the Hall voltage, enabling identification of higher harmonics and hysteretic switching behavior, as well as broadband nonlinear phenomena that would not be readily accessible with conventional lock‐in techniques.

We note that extrinsic thermoelectric effects^[^ ^14^, ^15^, ^26^ ^]^ as well as asymmetric scattering from magnon emission/absorption^[^ ^4^, ^5^ ^]^ may contribute to the nonlinear Hall signals discussed above, complicating the quantitative separation of each effect. However, none of these effects can cause the hysteresis observed at *B_x_* =  0.01 T because the estimated temperature always follows the current without delay, as shown in Figure S7 (Supporting Information). A more quantitative discussion of the relative contributions from the Nernst effect and magnetization reversal is provided in Note S5 (Supporting Information). In addition, while Joule‐heating‐induced magnetization reduction can contribute to the nonlinearity, this effect itself can generate only odd‐harmonic terms (see Note S4, Supporting Information). Therefore, the main contribution of the nonlinear signals, in particular the second‐harmonic signals, is magnetization dynamics, particularly in the case of *B_x_* =  0.01 T. We also provide a simple model calculation of magnetization reversal induced by AC current in Figure S9 (Supporting Information). The butterfly‐shaped *I_x_* − *V_y_* characteristic and the presence and magnitude of $V_{y}^{\prime \prime \left(n\right)}$ are consistent with the experiment. The calculation further shows that incorporating magnetization inhomogeneity and Joule‐heating‐induced magnetization reduction are important to reproduce the observations. For details of the calculation, see Note S6 (Supporting Information).

## 4 Asymmetric Frequency Mixing

**Figure 4:**
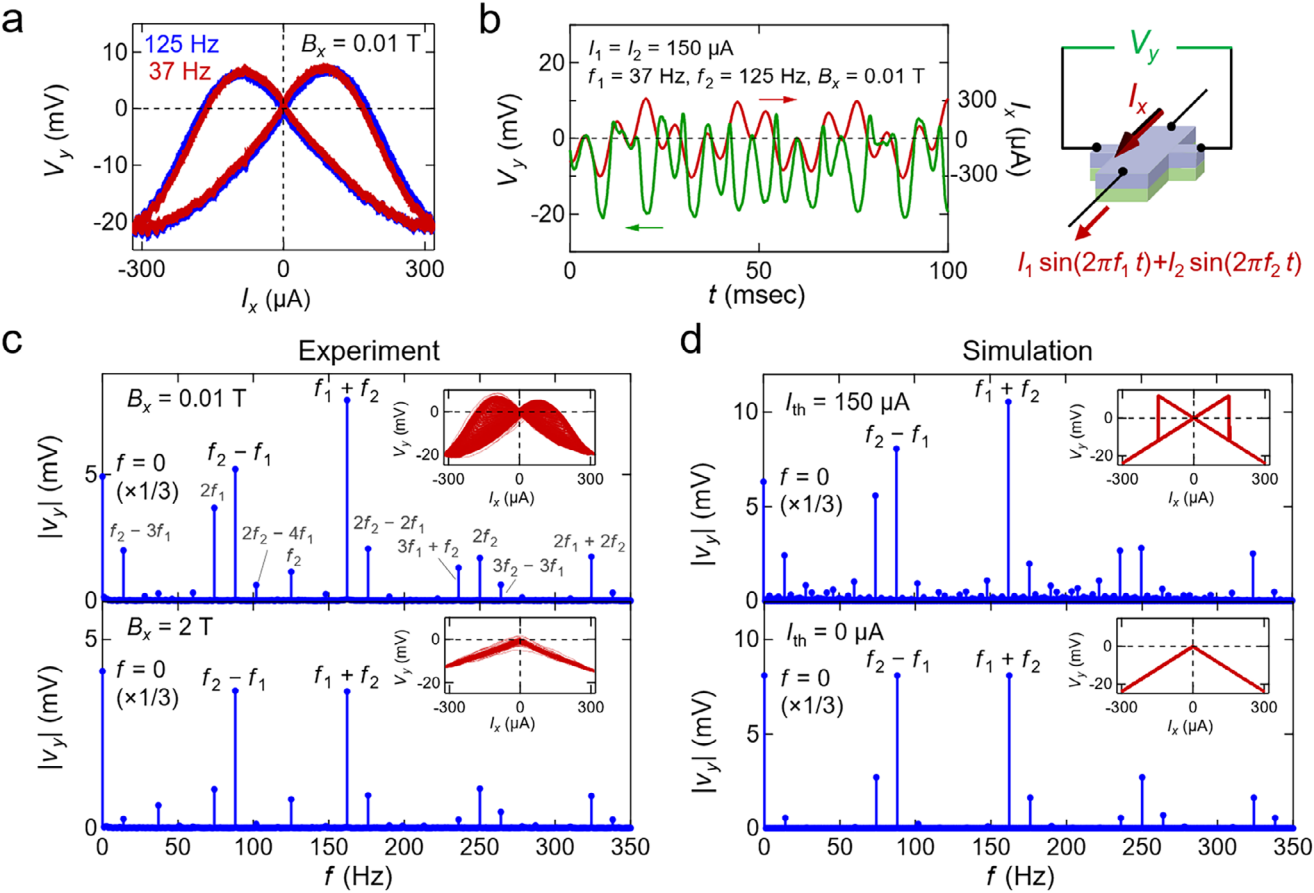
Frequency‐mixing accompanied by magnetization dynamics. a) Hall voltage versus current for the AC current with the frequencies, *f*_1_ =  37 Hz (red) and *f*_2_ =  125 Hz (blue), and the amplitude of 300 µ*A* under magnetic field *B_x_*  =  0.01 T and temperature *T*  =  2.5 K. b) The waveform of the input current including 2 frequency components *I_x_* (*t*) =  150sin (2π*f*_1_ *t*) + 150sin (2π*f*_2_ *t*) [µA] (red) and the Hall response *V_y_* (green) under magnetic field *B_x_*  =  0.01 T and temperature *T*  =  2.5 K. The schematic on the right side shows the experimental set‐up. c) Absolute value of Fourier component |*v_y_* (*f*)| from the measured responses under magnetic field *B_x_*  =  0.01 T (top) and *B_x_*  =  2 T (bottom). The inset shows Hall voltage versus current in each case. In the all panels of c and d, the peaks of the sum‐frequency *f*_1_ + *f*_2_ and the difference‐frequency |*f*_1_ − *f*_2_| is explicitly indicated. In the top panel for the experiment under 0.01 T, the peaks of other linear combinations (gray) are also indicated. d) Absolute value of Fourier component |*v_y_* (*f*)|, which is defined in the same way as c, from the simulated responses for *I*_th_ =  150 µA (top) and *I*_th_ =  0 µA (bottom), mimicking the experimental cases for *B_x_*  =  0.01 T and *B_x_*  =  2 T, respectively. All the data in c and d are plotted as a function of *f* with 1 Hz intervals.

Finally, we demonstrate a frequency‐mixing phenomenon^[^ ^27^ ^]^ enabled by AC current‐induced magnetization switching. In general, a nonlinear system driven by two frequencies *f*_1_ and *f*_2_ can generate signals at their sum *f*_1_ + *f*_2_ and difference |*f*_1_ − *f*_2_|. If the nonlinearity is purely polynomial in current, the amplitudes of the *f*_1_ + *f*_2_ and |*f*_1_ − *f*_2_| components are expected to be equal^[^ ^27^ ^]^ (see Note S7, Supporting Information). However, in our semi‐magnetic TI, the hysteresis of magnetization reversal breaks this symmetry. In the experiment, we apply *I_x_* (*t*)  = *I*_1_ sin (2π*f*_1_ *t*) + *I*_2_sin (2π*f*_2_ *t*) with *I*_1_ = *I*_2_  =  150 µA, *f*_1_ =  37 Hz, and *f*_2_ =  125 Hz. When only a single frequency (*f*_1_ =  37 Hz or *f*_2_ =  125 Hz) is applied (peak current 300 µA), the response is similar to our earlier single‐frequency result (**Figure** **4**a), confirming that the hysteretic behavior is governed by magnetization switching (Figure 1e,f), rather than the inertial dynamics of magnetic domains or capacitive/inductive elements of the electrical circuits. With both frequencies present (Figure 4b), we observe broadband wave mixing, ranging from DC (0 Hz) to 338 Hz in the Fourier spectrum of *V_y_* (Figure 4c). Notably, at a low magnetic field (0.01 T), the *f*_1_ + *f*_2_ component is substantially larger than the |*f*_1_ − *f*_2_| component. In contrast, at a high field (2 T), both peaks exhibit similar amplitudes, indicating a more conventional polynomial‐type nonlinearity. A numerical simulation (Figure 4d), assuming a simplified *I_x_* − *V_y_* characteristic with a well‐defined threshold (insets of Figure 4d), reproduces this asymmetry only when a finite threshold current *I*_th_ =  150 µA is considered (mimicking the low‐field case). If *I*_th_ =  0 µA (high‐field case), the two sidebands remain equal. Hence, the hysteretic magnetization reversal leads to asymmetric frequency mixing.

## 5 Conclusion

Our results show that an AC current can induce continuous magnetization reversal in a semi‐magnetic TI heterostructure, at a remarkably low current density (1.5 × 10^9^ A m^−2^). We clarify the nonlinear Hall effect that accompanies this process and demonstrate that the hysteretic, phase‐delayed Hall responses are governed by a threshold current for magnetization reversal; moreover, the applied magnetic field controls whether hysteresis appears. When a current containing components of two different frequencies is applied, we discover a distinctive frequency‐mixing effect where the magnitudes of the sum‐frequency and difference‐frequency components differ due to the hysteresis for magnetization reversal.

These electrically asymmetric frequency mixing may be exploited for selective extraction of desired frequency components in spintronic devices, with potential applications in photoacoustic imaging^[^ ^28^ ^]^ and microwave generation.^[^ ^29^ ^]^ The pronounced nonlinear Hall effect observed here also suggests opportunities for advanced signal‐processing functions such as Hall rectification^[^ ^30^, ^31^ ^]^ (including terahertz‐to‐DC conversion^[^ ^32^ ^]^ and AC‐to‐DC conversion^[^ ^33^ ^]^) and for neuromorphic hardware: elements that combine nonlinearity with short‐term memory are central to physical reservoir computing,^[^ ^34^, ^35^, ^36^, ^37^, ^38^, ^39^ ^]^ and our system has the potential for nonlinear transformation of inputs into higher‐dimensional outputs, suitable for a physical reservoir.

All measurements reported in this study were performed at 2.5 K. The use of ferromagnetic materials with higher Curie temperatures in the magnetic layer exchange‐coupled to the topological insulator could enable the operation even at room temperature.^[^ ^40^, ^41^, ^42^ ^]^ Although our present experiments are limited to low frequencies (≤10 kHz), likely due to circuit capacitance (Notes S2 and S5, Supporting Information), the intrinsic timescales of magnetization dynamics lie in the MHz‐GHz range (e.g., domain wall dynamics^[^ ^43^ ^]^ and coherent precession). Together with recent reports of spintronic rectification extending into the GHz‐THz range,^[^ ^44^, ^45^ ^]^ these considerations suggest that the nonlinear and hysteretic responses identified here may be extended to much higher frequencies in future studies. Thus, our study paves the way for harnessing hysteretic magnetization dynamics, with potential applications in spin–orbit‐based low‐power switching elements, and advanced nonlinear electronics such as neuromorphic computing.^[^ ^46^ ^]^

## 6 Experimental Section

### Sample Fabrication and Electric Transport Measurement

(Cr,Bi,Sb)_2_Te_3_/(Bi,Sb)_2_Te_3_ thin films were grown on InP(111) substrates by molecular beam epitaxy under the base pressure on the order of 10^−7^ Pa. The flux ratio *P*_Cr_: *P*_Bi_: *P*_Sb_:  *P*_Te_ =  1:9:16: 1000 was used to tune the Fermi energy inside the bulk gap. The films are fabricated into 10 µm wide Hall‐bar devices. The structures of them are illustrated in Figure S1a (Supporting Information). In a typical sample, the electrical transport properties using physical property measurement system (PPMS) were measured, as shown in Figure S1b (Supporting Information). The longitudinal and Hall resistance of a typical sample is about 10 and 2 kΩ, respectively. The Hall conductivity at the lowest temperature *T*  =  2.5 K is about 0.3 × *e* ^2^/*h*, which indicates the Fermi level is fairly close to the magnetization gap. From the temperature dependence of Hall and longitudinal resistivity, the Curie temperature can be determined as *T*_C _ ≈  50 K. The device‐to‐device variation of the experimental results are also shown in Figure S8 (Supporting Information).

### Time‐Domain Measurement

The Hall voltage as well as the longitudinal voltage in real time with an oscilloscope and a current source (Keithley 6221) was measured. The experimental setup in the PPMS chamber is shown in Figure S2 (Supporting Information). The oscilloscope monitors the Hall voltage *V_y_* (*t*) as

(3) $$V_{y}\left(t\right)=V_{\mathrm{CH}2}\left(t\right)-V_{\mathrm{CH}1}\left(t\right)$$

and the voltage on the resistor *V_R_* as

(4) $$V_{R}\;\left(t\right)=V_{\mathrm{CH}3}\;\left(t\right)$$

where *V*_CH1_(*t*), *V*_CH2_(*t*), and *V*_CH3_(*t*) are the measured voltages of the channels CH1, CH2, and CH3, respectively. It is noted that the current flowing through the circuit is obtained by

(5) $$I_{x}\left(t\right)=\frac{V_{R}\left(t\right)\left[V\right]}{1000\left[\Omega \right]}=\frac{V_{\mathrm{CH}3}\left(t\right)\left[V\right]}{1000\left[\Omega \right]}$$

## Conflict of Interest

The authors declare no conflict of interest.

## Author Contributions

Y.K. and M.M. contributed equally to this work. M.M. and Y.T. conceived the study. Y.K. and M.M. grew the samples with help of R.Y., A.T., and M.K. Y.K. and M.M. performed measurements with help of Y.F., Y.S., M.T.B., and M.K. All authors discussed the results. Y.K., M.M. and Y.T. wrote the manuscript with inputs from all other authors. Y.T. supervised the project.

## Supporting information



Supporting Information

## Data Availability

The data that support the findings of this study are available from the corresponding author upon reasonable request.
