# Supplementary material for: AC Current‐Driven Magnetization Switching and Nonlinear Hall Rectification in a Magnetic Topological Insulator
Source: Adv Mater. 2025 Oct 14;38(4):e06210. doi: 10.1002/adma.202506210 (PMC12810608; doi:10.1002/adma.202506210)
Supplement: Supplementary file 1 — Supporting Information [file ADMA-38-e06210-s001.pdf]

ADVANCED MATERIALS

Supporting Information

for *Adv. Mater.*, DOI 10.1002/adma.202506210

AC Current-Driven Magnetization Switching and Nonlinear Hall Rectification in a Magnetic Topological Insulator

Yuto Kiyonaga, Masataka Mogi, Ryutaro Yoshimi, Yukako Fujishiro, Yuri Suzuki, Max T. Birch, Atsushi Tsukazaki, Minoru Kawamura, Masashi Kawasaki and Yoshinori Tokura*

Supplementary Information for AC Current-Driven Magnetization Switching and Nonlinear Hall Rectification in a Magnetic Topological Insulator

Yuto Kiyonaga,† Masataka Mogi,†, Ryutaro Yoshimi, Yukako Fujishiro, Yuri Suzuki, Max T. Birch, Atsushi Tsukazaki, Minoru Kawamura, Masashi Kawasaki and Yoshinori Tokura*

Y. Kiyonaga, M. Mogi, Y. Suzuki, A. Tsukazaki, M. Kawasaki, Y. Tokura
Department of Applied Physics and Quantum-Phase Electronics Center (QPEC)
The University of Tokyo, Tokyo 113-8656, Japan
Email: mogi@ap.t.u-tokyo.ac.jp

R. Yoshimi, Y. Fujishiro, M. T. Birch, M. Kawamura, M. Kawasaki, Y. Tokura
RIKEN Center for Emergent Matter Science (CEMS)
RIKEN, Wako 351-0198, Japan

R. Yoshimi
Department of Advanced Materials Science
The University of Tokyo, Kashiwa 277–8561, Japan

Y. Fujishiro
RIKEN Cluster for Pioneering Research (CPR)
RIKEN, Wako 351-0198, Japan

Y. Tokura
Tokyo College
The University of Tokyo, Tokyo 113-8656, Japan

†Y. Kiyonaga and M. Mogi contributed equally to this work.

Supplementary Note 1: Analysis of the raw data in the time-domain measurements

Using an oscilloscope, we measured Hall voltage in response to current in the time domain. To exclude the background from longitudinal voltage due to electrode misalignment, we subtracted the measured data under the magnetic field in the $-x$ direction from the one under that in the $+x$ direction (anti-symmetrization). Here we emphasize that when measuring data under the opposite field, the way of initialization is also inverted. For example, we measured $V_y(t)$ under $B_x = +0.01$ T after initializing magnetization by setting to $B_x = +2$ T, and then measured $V_y(t)$ under $B_x = -0.01$ T after setting to $B_x = -2$ T. When both the initializing field and the assisting field are inverted, the Hall voltage signal originating from the initial magnetization and magnetization dynamics is inverted, while the background from the longitudinal resistance remains unchanged. Therefore, this anti-symmetrization method is effective.

Here, we show the data before and after anti-symmetrization for the cases of pulse (Figure S3) and AC (Figure S4). In the case of pulses, the probe current is small (10 μA), which suppresses temperature rise, allowing behaviors such as the sign change in the Hall resistance associated with magnetization reversal to be observed even before anti-symmetrization. On the other hand, in the case of AC, the background due to longitudinal resistance is significant relative to the Hall voltage signal, the anti-symmetrization procedure is necessary for the magnetization reversal behavior to be observed. In both cases, however, the behavior is similar: when the current flows in the $+x$ direction, the magnetization points in the $-z$ direction, and when the current flows in the $-x$ direction, the magnetization points in the $+z$ direction.

Supplementary Note 2: Frequency dependence of the magnetization reversal

We mainly use $I_x(t) = I_0 \sin(2\pi f t)$ with $I_0 = 300$ μA and f=101 Hz as the input current in the main text. Here we show in Figure S5 the response waveforms $V_y(t)$ and $I_x - V_y$ curves and $I_x - R_{yx}$ curves for various frequencies $f = 11, 101, 1001, 10001$ Hz. Overall, the qualitative behavior is basically independent of frequency for lower frequencies $f = 11, 101, 1001$ Hz. At the highest frequency $f = 10001$ Hz, the current is attenuated by the parasitic capacitance parallel to the sample, which causes a reduction in the current amplitude and a trivial phase change between current and Hall voltage. Therefore, at such a high frequency, it is not possible to correctly measure the Hall voltage as the response to the current in the time domain.

The frequency dependence of the responses presented here is attributed to the parasitic capacitance because the Hall voltage when the current is $I_x = 0$ was not $V_y = 0$ at the higher frequency. However, mechanisms other than parasitic capacitance through which the response depends on frequency, such as the inertia of the magnetization dynamics to the current, remain elusive. To explore such intrinsic frequency dependence, improvements in the equipment are necessary to enable high-frequency measurements without the influence of parasitic capacitance.

Supplementary Note 3: Reproducibility of AC-driven magnetization switching across devices

We have also conducted similar measurements on other samples and successfully reproduce the results qualitatively in all devices (Device #1, #2, and #3), which were fabricated from different films but the same stacking structure. Although the Hall resistance measured at a low excitation current (~ 1 μA) (Figure S6, top) shows slight variation from device to device, the butterfly-shaped $I_x - V_y$ characteristics showing AC current-induced magnetization switching is reproducible, as shown in Figure S6, middle and bottom.

We note, however, that Devices #2 and #3 exhibit an asymmetry in V_y between $I_x = I_{peak}$ and $I_x = -I_{peak}$. This asymmetry likely originates from a slight misalignment of the sample against the lateral magnetic field, and the fact that the magnetization does not fully reverse by current. In the presence of magnetic anisotropy, such misalignment can cause an imbalance between up and down domains during initialization, which is then reflected in the asymmetry of the switching behavior.

In contrast, for Device #1 (mainly used for the time-domain measurements), we carefully adjusted the alignment by first measuring the angular dependence of the Hall resistance to identify the orientation where the signal is maximized, and then refining the angle step by step until the peak became symmetric. After this procedure, the sample angle was fixed and not changed throughout the measurements. This alignment ensured that the results for Device #1 were almost free from the asymmetry observed in Devices #2 and #3.

Supplementary Note 4: Estimation of temperature increase caused by Joule heating

We attribute the hysteretic behavior to the magnetization reversal caused by spin-orbit torque. However, there would be still another explanation that the temperature would show a hysteretic change because of Joule heating and accordingly the Hall response would reflect it. To exclude this possibility, we estimate the temperature variation during applying current using longitudinal resistivity as a thermometer. We first measured the temperature dependence of the longitudinal resistance R_{xx} under the out-of-plane magnetic field $B_z = 1$ T (Figure S7a), which is large enough to fix the magnetization along *z* direction even under a large current excitation. The sensing current is as low as $I = 0.1$ μA, enabling us to ignore Joule heating. We then measured Hall resistance under AC with the amplitude of 300 μA and the frequency of 101 Hz as shown in the middle panel of Figure S7b. At each time, we can estimate the sample temperature from the value of R_{xx} as shown on the lower side. There is certainly no hysteresis in temperature, and it does not exceed the critical temperature $T_\mathrm{C} = 50$ K. This reinforces the idea that magnetization reversal occurs due to spin-orbit torque and that the hysteresis in the Hall voltage is caused by magnetization reversal.

In addition to ruling out such hysteretic heating effects, we also consider the possible thermal contribution to nonlinear Hall responses through the reduction of magnetization due to increased temperature. A rise in sample temperature due to Joule heating would reduce the anomalous Hall resistance, which can be approximated as $R_{yx} \approx R_{yx}^0 - \alpha\Delta T(t)$. Since the heating power scales with ${I_x}^2(t)$ without delay (i.e., no phase shift, see Figure S7), the resulting temperature variation is $\Delta T(t) \propto {I_x}^2(t) = \frac{1}{2} I_0^2(1 - \cos(2\omega t))$, which contains only a dc component and a component at frequency 2*f*. Consequently, the Hall voltage $V_y(t) = R_{yx}(t)I_x(t) \approx \{R_{yx}^0 - \alpha T(t)\}I_x(t)$ produces only odd harmonics $(2n + 1)f$, and therefore cannot produce the second harmonic signal observed in our experiments.

Supplementary Note 5: Lock-in measurements of second-harmonic Hall responses

To confirm the nonlinear response observed in the time-domain measurement, we performed lock-in measurements of second-harmonic Hall responses under AC current with the same frequencies, amplitudes and temperature conditions.

Figure S8a,b shows the magnetic field B_x dependence for current amplitudes of 14, 71, 141, and 300 μA at a frequency of 101 Hz. The lock-in measurements consistently show a giant second-harmonic component $R'^{(2)}_{yx}$ and a phase-shifted component $R''^{(2)}_{yx}$. A sharp peak appears in the low-field region around $B_x = 0.05$ T at high current amplitudes ($I_{\text{peak}} = 141, 300$ μA), reflecting the enhanced nonlinear Hall effect arising from AC magnetization switching. This feature is absent at low current amplitude ($I_{\text{peak}} = 14$ μA).

At higher magnetic fields, on the other hand, $R'^{(2)}_{yx}$ exhibits a field-induced enhancement even though magnetization switching does not occur. Two possible origins of this behavior can be considered: small magnetization oscillation driven by SOT (as discussed in the main text) and the anomalous and/or normal Nernst effect due to a temperature gradient caused by Joule heating. The former mechanism should be suppressed at higher magnetic fields, whereas the latter does not suppress the signal by magnetic fields because it reflects the in-plane magnetization and Lorentz force. Since the value of $R'^{(2)}_{yx}$ in this region is much smaller than the peak value at low B_x, we conclude that the Nernst effect is not a dominant contribution, less than$R^{(2)}_{yx}(B_x = 9\text{ T})/R^{(2)}_{yx,\text{peak}} \sim 40\%$ even under a maximal overestimation.

The current amplitude dependence also provides useful insight into thermal effects, which are expected to increase with current. In the main text, the maximum current amplitude is 300 μA, but we have also carried out measurements at higher amplitudes. For amplitudes above 300 μA, both $R'^{(2)}_{yx}$ and $R''^{(2)}_{yx}$ become smaller. This reduction arises because the temperature rising due to Joule heating weakens the magnetization and thereby reduces the nonlinear Hall response. The detailed current-amplitude dependence of second-harmonic Hall signals are shown in Figure S8e. These results indicate that the Joule heating suppresses the signal rather than contributes to it, which is inconsistent with an interpretation that the thermal effects are the dominant origin of the observed behavior.

In Figure S8f, we further present the frequency dependence of the second harmonic Hall signal taken at a low field (+0.05 T), where AC-current-induced magnetization switching occurs. The response remains nearly constant up to 500 Hz, starts to decrease above this frequency, and vanishes above 10 kHz. We attribute this behavior to the parasitic capacitance in our measurement circuit, which decreases the amplitude and shifts the phase against the input current, as also inferred in Figure S5. If measurements were extended to sufficiently high frequencies, intrinsic factors such as magnetic domain wall dynamics could also become relevant. The typical domain wall velocity driven by current is on the order of 100 m/s (e.g., permalloy[S1]), corresponding to a timescale of $100\,\mathrm{ns}$, i.e., $10\,\mathrm{MHz}$ in frequency. Thus, intrinsic magnetization dynamics may affect in the MHz regime, while our present measurements are limited by extrinsically by the setup.

Supplementary Note 6: Simple model calculation of magnetization dynamics

We conducted numerical simulations using simple models that either neglect or include the magnetization inhomogeneity and the reduction of magnetization due to Joule heating, and find that our results are reasonably reproduced when both factors are included. In the following, we show the results of the respective cases; Simulation 1, 2 and 3 with different assumptions.

Simulation 1: Without reduction of magnetization due to Joule heating or magnetic domain inhomogeneity

(1) In the initial state, magnetic domains with opposite out-of-plane magnetization directions cancel each other, resulting in zero net magnetization.

(2) Magnetization flips to $M_z = -M_0$ (corresponding to $R_{yx} = -150\ \Omega$; The value $150\ \Omega$ corresponds to the anomalous Hall resistance of the magnetization fraction that responds to current-induced switching as determined from Figure 1e.) once the current exceeds the threshold value $I = I_{\mathrm{th}}$. Here, we set $I_{\mathrm{th}} = 150$ μA.

(3) Once flipped, the magnetization remains pinned along the $-z$ direction until current decreases down to $I = -I_{\mathrm{th}}$. At this point, it switches back from $M_z = -M_0$ to $M_z = M_0$ (and from $R_{yx} = -150\ \Omega$ to $R_{yx} = 150\ \Omega$).

Simulation 2: Without reduction of magnetization due to Joule heating and with magnetic domain inhomogeneity

(1) Same as Simulation 1(1).

(2) Each domain is assumed to have a different threshold current for switching. The distribution of threshold values is modeled by a Gaussian distribution function centered at $\langle I_{\mathrm{th}} \rangle = 150$ μA;

$$\frac{1}{\sqrt{\pi}\sigma_{I_{\mathrm{th}}}} \exp\left(-\frac{(I_{\mathrm{th}} - \langle I_{\mathrm{th}} \rangle)^2}{2\sigma_{I_{\mathrm{th}}}{}^2}\right),$$

where $\sigma_{I_{\mathrm{th}}}$ denotes the standard deviation of the threshold values. Here we set $\sigma_{I_{\mathrm{th}}} = 150$ μA, which yields an effective distribution ranging from $I_{\mathrm{th}} = 0$ μA to $I_{\mathrm{th}} = 300$ μA.

For numerical simulations, the continuous distribution of the threshold current is discretized into small intervals of width ΔI_{th}. Domains with threshold values within a given interval $I_{\mathrm{th}} < I < I_{\mathrm{th}} + \Delta I_{\mathrm{th}}$ are assumed to switch collectively when the applied current $I(t)$ crosses this range. Consequently, their contribution to the anomalous Hall resistance is expressed as

$$R_{yx} = -150\ \Omega \times \frac{\Delta I_{\mathrm{th}}}{\sqrt{\pi}\sigma_{I_{\mathrm{th}}}} \exp\left(-\frac{(I_{\mathrm{th}} - \langle I_{\mathrm{th}} \rangle)^2}{2\sigma_{I_{\mathrm{th}}}{}^2}\right).$$

The interval ΔI_{th} is an arbitrary constant introduced to discretize the distribution, and we set $\Delta I_{\text{th}} = 1.5$ μA in the calculation.

(3) Once the magnetization of each domain has flipped, it remains pinned and does not flip back until the current decreases into the interval $-(I_{\text{th}} + \Delta I_{\text{th}}) < I < -I_{\text{th}}$. At this point, the magnetization is flipped back, and their contribution to the anomalous Hall resistance is given by $R_{yx} = 150\ \Omega \times \frac{\Delta I_{\text{th}}}{\sqrt{\pi}\sigma_{I_{\text{th}}}} \exp\left(-\frac{(I_{\text{th}} - \langle I_{\text{th}} \rangle)^2}{2\sigma_{I_{\text{th}}}{}^2}\right)$.

(4) Here we neglect the reduction of anomalous Hall resistance due to temperature rising, thus the Hall voltage is

$$V_y(t) = \sum_{\Delta I_{\text{th}}} I(t) \times 150 \times \frac{\Delta I_{\text{th}}}{\sqrt{\pi}\sigma_{I_{\text{th}}}} \exp\left(-\frac{(I_{\text{th}} - \langle I_{\text{th}} \rangle)^2}{2\sigma_{I_{\text{th}}}{}^2}\right)$$

at each time t.

Simulation 3: With reduction of magnetization due to Joule heating and magnetic domain inhomogeneity

(1) Same as Simulation 1(1).

(2) Same as Simulation 2(2).

(3) Same as Simulation 2(3).

(4) To incorporate the temperature increase, we approximate the effective anomalous Hall resistance as $R_{yx}(T) \approx \frac{50-T}{50} R_{yx}(T = 0)$, so that it decreases linearly with increasing T and vanishes at $T_{\text{C}} \approx 50$ K (see Figure S1b). The temperature rise can be estimated from Figure S7 and is approximately given by $T \approx 25\ \text{K} \times \frac{|I|\ (\mu\text{A})}{300\ \mu\text{A}}$.

Accordingly, the Hall voltage at each time t is expressed as

$$V_y(t) = \sum_{\Delta I_{\text{th}}} I(t) \times \frac{50 - 25 \times \frac{|I(t)|}{300}}{50} \times 150 \times \frac{\Delta I_{\text{th}}}{\sqrt{\pi}\sigma_{I_{\text{th}}}} \exp\left(-\frac{(I_{\text{th}} - \langle I_{\text{th}} \rangle)^2}{2\sigma_{I_{\text{th}}}{}^2}\right).$$

The waveform, characteristic, and Fourier components calculated using the above model are shown in Figure S9a-c, where they are compared with the experimental data presented in Figure 3 of the main text. By considering both the reduction of anomalous Hall resistivity due to Joule heating and the inhomogeneous magnetic domains (i.e., Simulation 3), we successfully reproduce the experimental waveform quantitatively. This supports our conclusion that the nonlinear Hall response is primarily caused by magnetization switching induced by AC current.

Supplementary Note 7: Nonlinear Hall effect (polynomial-type)

Here, we consider the Hall voltage $V_y(t)$ in response to the current

$$I_x(t) = I_0 \sin(\omega t)$$

when V_y is written as a power series of I_x;

$$V_y = R_{yx} I_x + R_{yxx} I_x^2 + R_{yxxx} I_x^3 + R_{yxxxx} I_x^4 + \cdots$$

where $R_{y\overbrace{xx\cdots x}^{n}}$ $(n = 1, 2, 3, 4, \cdots)$ is coefficient of each order of I_x. Substituting the $I_x(t)$ into this, we obtain

$$V_y(t) = R_{yx} I_0 \sin(\omega t) + R_{yxx}(I_0 \sin(\omega t))^2 + R_{yxxx}(I_0 \sin(\omega t))^3 + R_{yxxxx}(I_0 \sin(\omega t))^4 + \cdots$$

$$= R_{yx} I_0 \sin(\omega t) + R_{yxx} I_0^2 \frac{1 - \cos(2\omega t)}{2} + R_{yxxx} I_0^3 \left(\frac{3}{4}\sin(\omega t) - \frac{1}{4}\sin(3\omega t)\right)$$

$$+ R_{yxxxx} I_0^4 \left(\frac{3}{8} - \frac{1}{2}\cos(2\omega t) + \frac{1}{8}\cos(4\omega t)\right) + \cdots$$

$$= V_y^{(0)} + V'^{(1)}_y \sin(\omega t) + V'^{(2)}_y \cos(2\omega t) + V'^{(3)}_y \sin(3\omega t) + V'^{(4)}_y \cos(4\omega t) + \cdots,$$

where

$V_y^{(0)} = I_0^2 R_{yxx}/2 + 3I_0^4 R_{yxxxx}/8 + \cdots,$

$V'^{(1)}_y = I_0 R_{yx} + 3I_0^4 R_{yxxxx}/4 + \cdots, V'^{(2)}_y = -I_0^2 R_{yxx}/2 - I_0^4 R_{yxxxx}/2 + \cdots,$

$V'^{(3)}_y = -I_0^3 R_{yxxx}/4 + \cdots, V'^{(4)}_y = I_0^4 R_{yxxxx}/8 + \cdots.$

In this way, all the odd-harmonic components appear as sine functions, while all the even harmonic components appear as cosine functions in this power series, as mentioned in the main text.

Supplementary Note 8: Frequency-mixing effect with and without threshold

In the main text, we discuss the frequency-mixing effect using the current including 2 frequencies $f_1 = 37$ Hz and $f_2 = 125$ Hz. Here we describe how the mixed frequencies appear in the response.

First, let the current be $I_x(t) = I_1\sin(2\pi f_1 t) + I_2\sin(2\pi f_2 t)$. Then, we assume that the Hall voltage can be written as a power series of I_x;

$$V_y(t) = R_{yx}I_x(t) + R_{yxx}(I_x(t))^2 + R_{yxxx}(I_x(t))^3 + R_{yxxxx}(I_x(t))^4 + \cdots$$

where $R_{y\overbrace{xx\cdots x}^{n}}$ $(n = 1, 2, 3, 4, \cdots)$ is coefficient of each order of $I_x(t)$. Substituting $I_x(t)$ into it, we obtain a lot of components with frequencies which are expressed as linear combinations of f_1 and f_2. For example, the second order term is written as below;

$$\begin{aligned}
R_{yxx}(I_x(t))^2 &= R_{yxx}(I_1\sin(2\pi f_1 t) + I_2\sin(2\pi f_2 t))^2 \\
&= R_{yxx}\left(I_1^2\frac{1-\cos(4\pi f_1 t)}{2} + I_2^2\frac{1-\cos(4\pi f_2 t)}{2} \right. \\
&\quad \left. + I_1I_2\{\cos[2\pi(f_2 - f_1)t] - \cos[2\pi(f_1 + f_2)t]\}\right) \\
&= R_{yxx}\frac{I_1^2 + I_2^2}{2} - \frac{R_{yxx}I_1^2}{2}\cos(4\pi f_1 t) - R_{yxx}\frac{R_{yxx}I_2^2}{2}\cos(4\pi f_2 t) \\
&\quad + R_{yxx}I_1I_2\cos[2\pi(f_2 - f_1)t] - R_{yxx}I_1I_2\cos[2\pi(f_1 + f_2)t]
\end{aligned}$$

In this way, the sum- and difference-frequency components are derived. Other linear combinations of the form, $af_1 + bf_2$ (a and b being integer numbers), are also derived from higher order terms.

Here, we prove that the components with frequencies $af_1 + bf_2$ and $af_1 - bf_2$ have the same amplitude if $V_y(t)$ is written in a purely polynomial form of current without a threshold behavior. First, the components of $af_1 + bf_2$ and $af_1 - bf_2$ comes only from the terms of

$$[I_1\sin(2\pi f_1 t)]^{a+2m}[I_2\sin(2\pi f_2 t)]^{b+2n} \ (m, n = 0, 1, 2, \cdots),$$

when f_1 and f_2 are coprime like 37 Hz and 125 Hz. Because the current can be also expressed as $I_x(t) = I_1\sin2\pi f_1 t - I_2\sin2\pi(-f_2)t$, this term should be equal to

$$\begin{aligned}
[I_1\sin(2\pi f_1 t)]^{a+2m}[I_2\sin(2\pi f_2 t)]^{b+2n} &= [I_1\sin(2\pi f_1 t)]^{a+2m}[-I_2\sin(-2\pi f_2 t)]^{b+2n} \\
&= (-1)^b[I_1\sin(2\pi f_1 t)]^{a+2m}[I_2\sin(-2\pi f_2 t)]^{b+2n} \ (m, n = 0, 1, 2, \cdots).
\end{aligned}$$

Thus, the coefficients $C_{a,b}$ of the term of $\cos[2\pi(af_1 + bf_2)t]$ (or $\sin[2\pi(af_1 + bf_2)t]$ and $C_{a,-b}$ of $\cos[2\pi(af_1 - bf_2)t]$ (or $\sin[2\pi(af_1 - bf_2)t]$) are necessarily connected by

$$C_{a,b} = (-1)^b C_{a,-b}$$

This shows that the amplitudes of the two components are equal, in accord with the observation as shown in the case of B_x = 2 T in Fig. 4d of the main text.

Supplementary Note 9: Estimation of the magnetic anisotropy

We estimate the magnetic anisotropy energy by evaluating the in-plane and out-of-plane magnetization, M_x and M_z, as a function of the in-plane magnetic field B_x. These are obtained from the relations, $M_x \propto \sqrt{(R_{yx}(B_x = 0))^2 - (R_{yx}(B_x))^2}$ and $M_z \propto R_{yx}(B_x)$, which indicate that the magnetization, initially oriented along the out-of-plane (z) direction, gradually tilts toward the in-plane (x) direction. The anisotropy field, defined as the magnetic field required to fully align the magnetization along the x-direction, is estimated to be about 0.2 T.

Since the magnetization in our sample comes from the Cr atoms, magnetization per magnetic ion (Cr^{3+}) is $3\mu_B$. With the Cr doping level of 4% and the unit cell volume of $(Bi_{0.36}Sb_{0.64})_2Te_3$ estimated to be $0.64 \times 0.469\ \mathrm{nm}^3 + 0.36 \times 0.509\ \mathrm{nm}^3 = 0.483\ \mathrm{nm}^3$, the anisotropy energy per volume is $6 \times 0.04 \times 17\ \mu\mathrm{eV}/0.483\ \mathrm{nm}^3 = 1.3\ \mathrm{kJ/m}^3$. which is smaller than typical soft magnetic materials (e.g., FeTaC with $20\ \mathrm{kJ/m}^3$ [S2]). This softness of magnetization may play a role in an efficient switching by relatively small current.

References

[S1] Hayashi, M. et al. Current Driven Domain Wall Velocities Exceeding the Spin Angular Momentum Transfer Rate in Permalloy Nanowires, *Phys. Rev. Lett.* **98**, 037204 (2007).

[S2] Samantaray, B. et al. Spin dynamics and frequency dependence of magnetic damping study in soft ferromagnetic FeTaC film with a stripe domain structure, *AIP Advances* **5**, 067157 (2015).

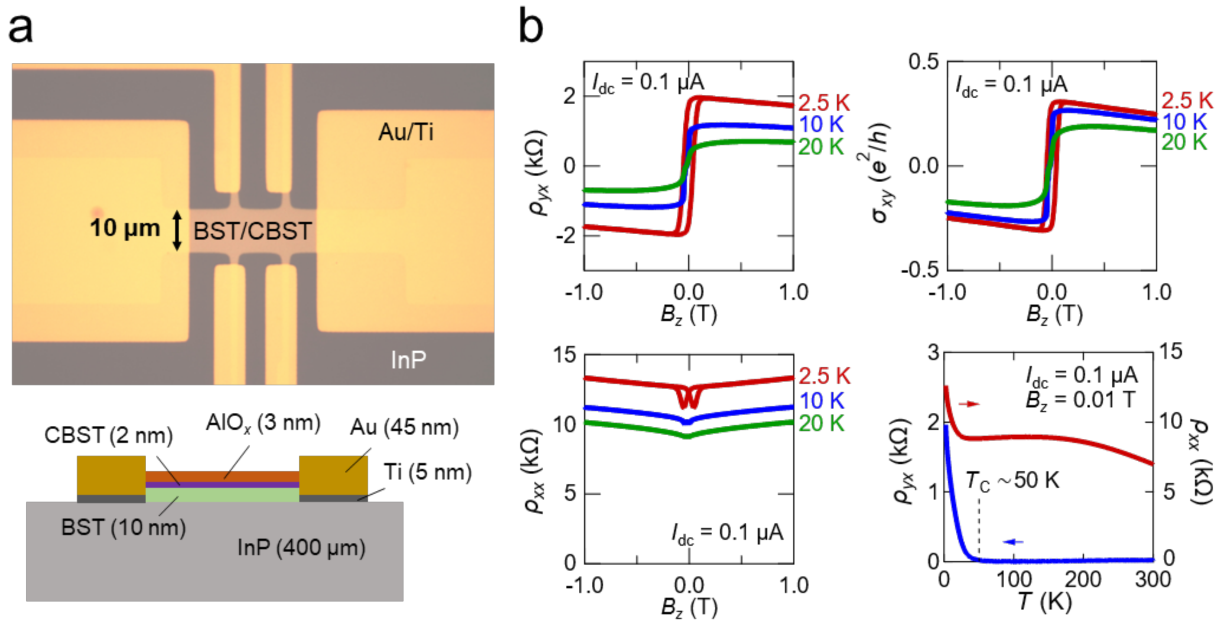

Figure S1: Device geometry and basic transport properties. a Optical microscope image of the representative device used in our experiment (top) and the cross-sectional schematic of the device in detail (bottom). $(Bi,Sb)_2Te_3/(Cr,Bi,Sb)_2Te_3$ heterostructure is grown on InP(111) substrate. After depositing the AlO_x capping layer, the samples were patterned into the Hall-bar device with the Au/Ti electrodes. **b** Fundamental transport properties of the sample (Hall-bar device) measured by low DC current $I_{\mathrm{dc}} = 0.1$ μA using PPMS. The upper left, the upper right, and the lower left panels show magnetic field (B_z) dependence of Hall resistivity ρ_{yx}, Hall conductivity σ_{xy}, and longitudinal resistivity ρ_{xx}, respectively. All of them are measured at temperatures 2.5 K (red), 10 K (blue), 20 K (green). Temperature dependence of ρ_{xx} (red) and ρ_{yx} (blue) under low magnetic field $B_z = 0.01$ T are presented in the lower right panel. From the rise of the anomalous Hall resistivity (ρ_{yx} at 0.01T), the transition temperature is determined as $T_{\mathrm{C}}{\sim}50$ K.

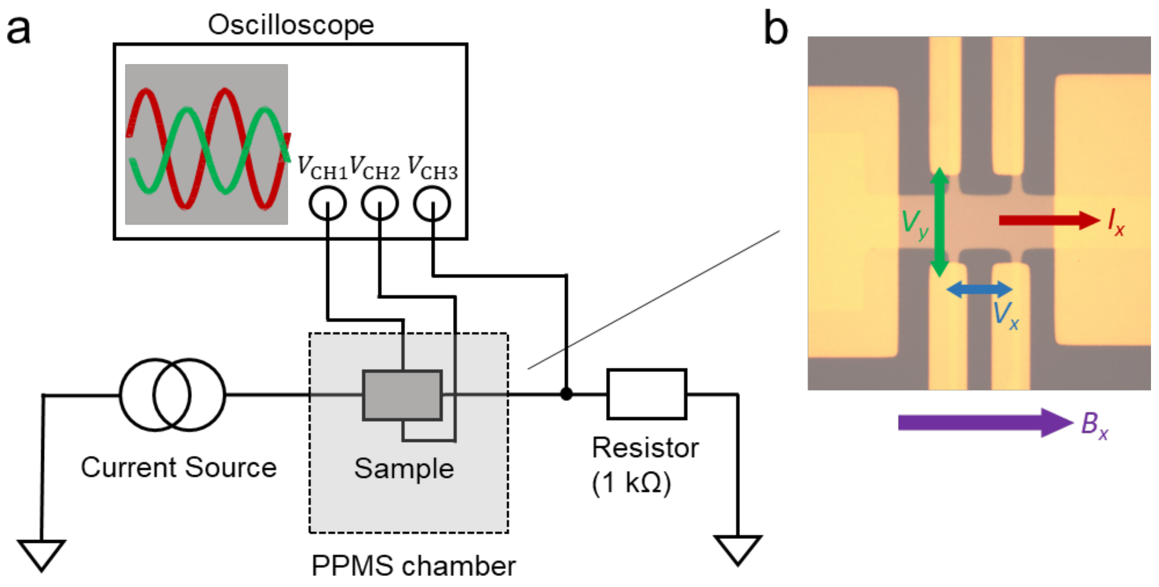

Figure S2: Time-domain measurement setup. a Circuit diagram of the measurement system. Current from the current source flows through the sample and resistor connected in parallel. The oscilloscope measures the Hall voltage or longitudinal voltage of the sample while simultaneously monitoring the current flowing through the system from the voltage across the resistor. The sample is put in a PPMS chamber. **b** Configuration of the current (red), the Hall voltage (green), the longitudinal voltage (blue), and the magnetic field (purple) in the sample. The shape of the sample is the same as in Figure S1**a**.

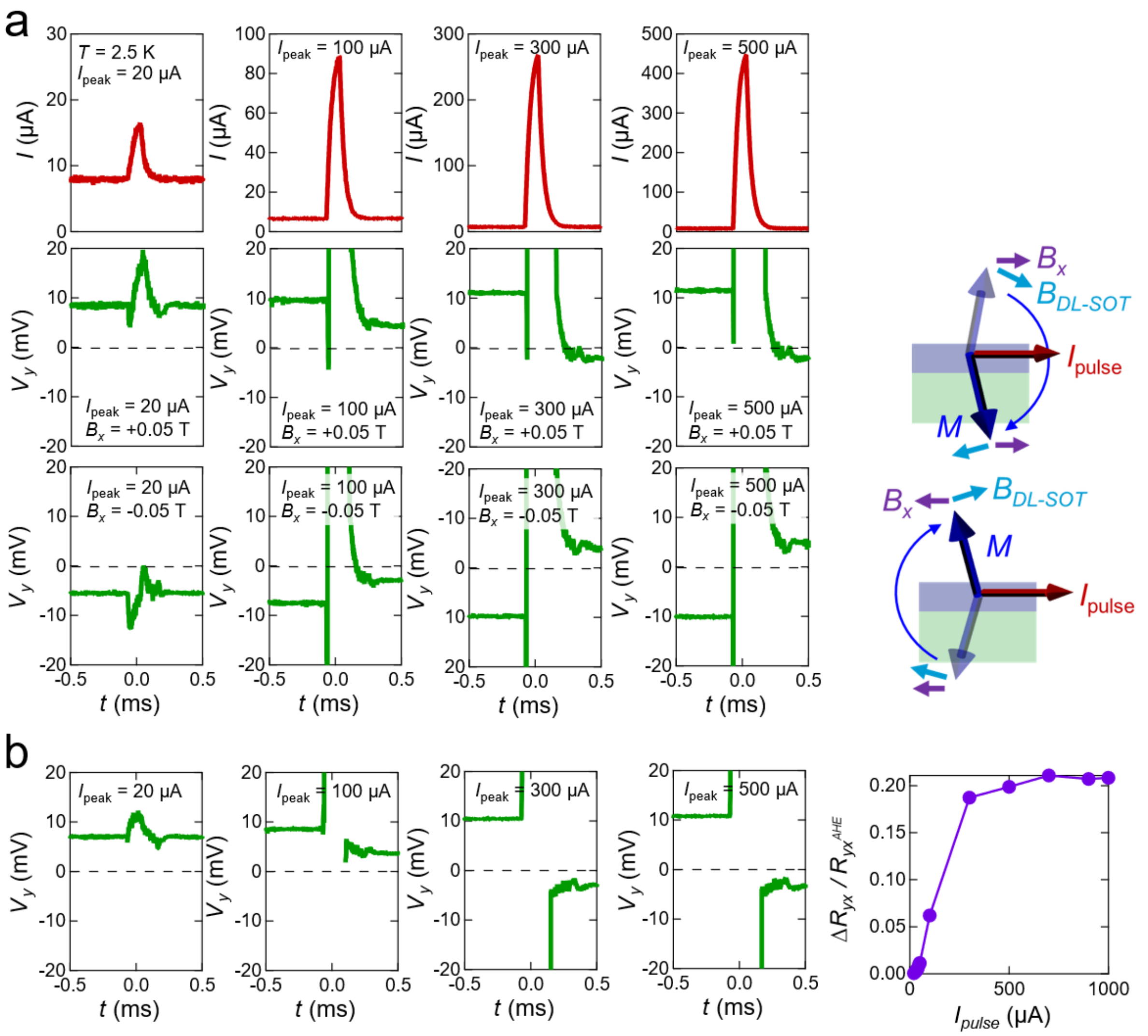

Figure S3: Raw data and anti-symmetrized data of the time-domain measurements of magnetization reversal induced by pulse. a Raw data of Hall voltage (green line in the middle section) in response to pulse current (red, upper) for the peak current values $I_{\text{peak}} = 20, 100, 300, 500\ \mu\text{A}$. The middle and lower sections show the responses under the in-plane magnetic field of $+0.05$ T and -0.05 T, respectively. The configurations of the magnetic field (purple arrow), the effective magnetic field of damping-like SOT (light blue), the magnetization (blue), and the pulse current (red) are illustrated on the right side. **b** Anti-symmetrized responses obtained from the raw data (shown in **a**) for ± 0.05 T. The peak value dependence of the magnetization switching ratio is also shown in the right side. Through this figure, a different sample was used for the measurement, compared to the one used in the other figures.

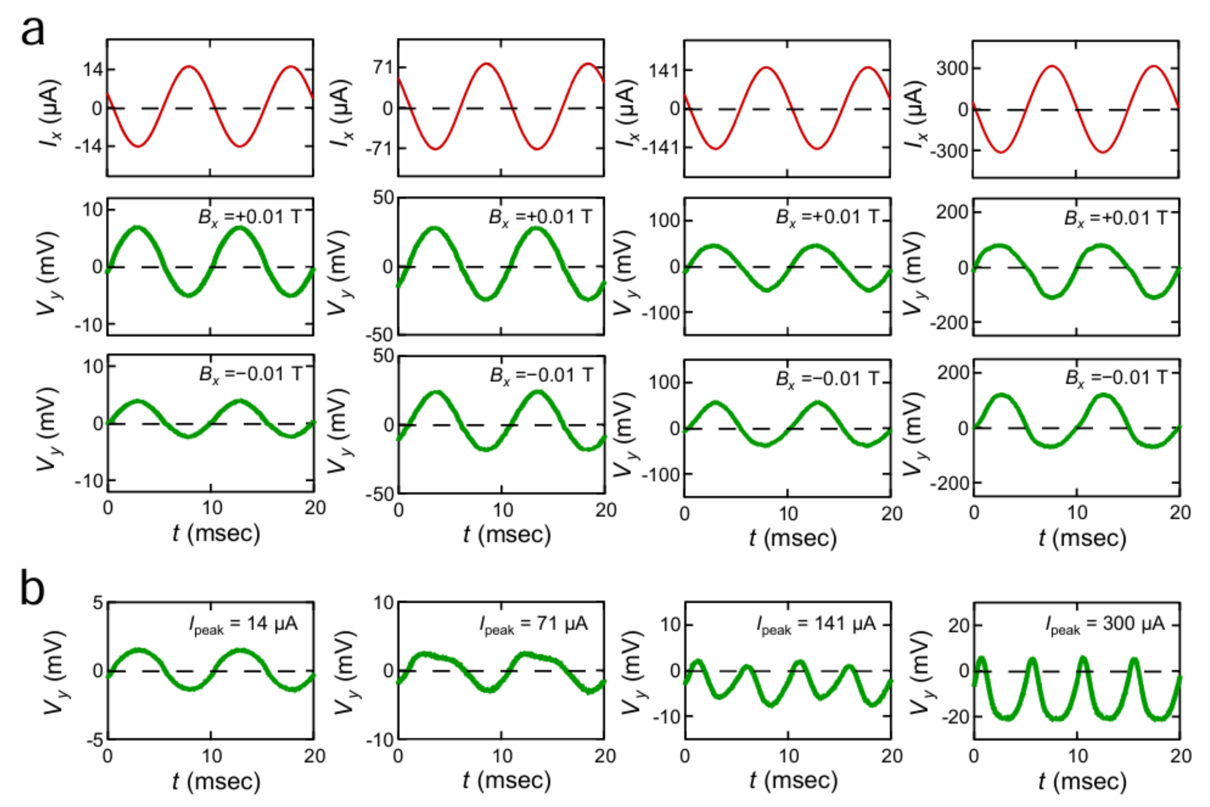

Figure S4: Raw data and anti-symmetrized data of the time-domain measurements of magnetization reversal induced by AC. a Raw data of Hall voltage (green lines in the middle and lower sections) in response to AC current (red, upper) for the peak current values $I_{\text{peak}} = 14, 71, 141, 300$ μA. The middle and lower sections show the responses under the in-plane magnetic field of $+0.01$ T and -0.01 T, respectively. **b** Anti-symmetrized data obtained from the raw data (shown in **a**) for ± 0.01 T. Increasing I_{peak}, the response $V_y(t)$ gradually becomes nonlinear, as discussed in the main text.

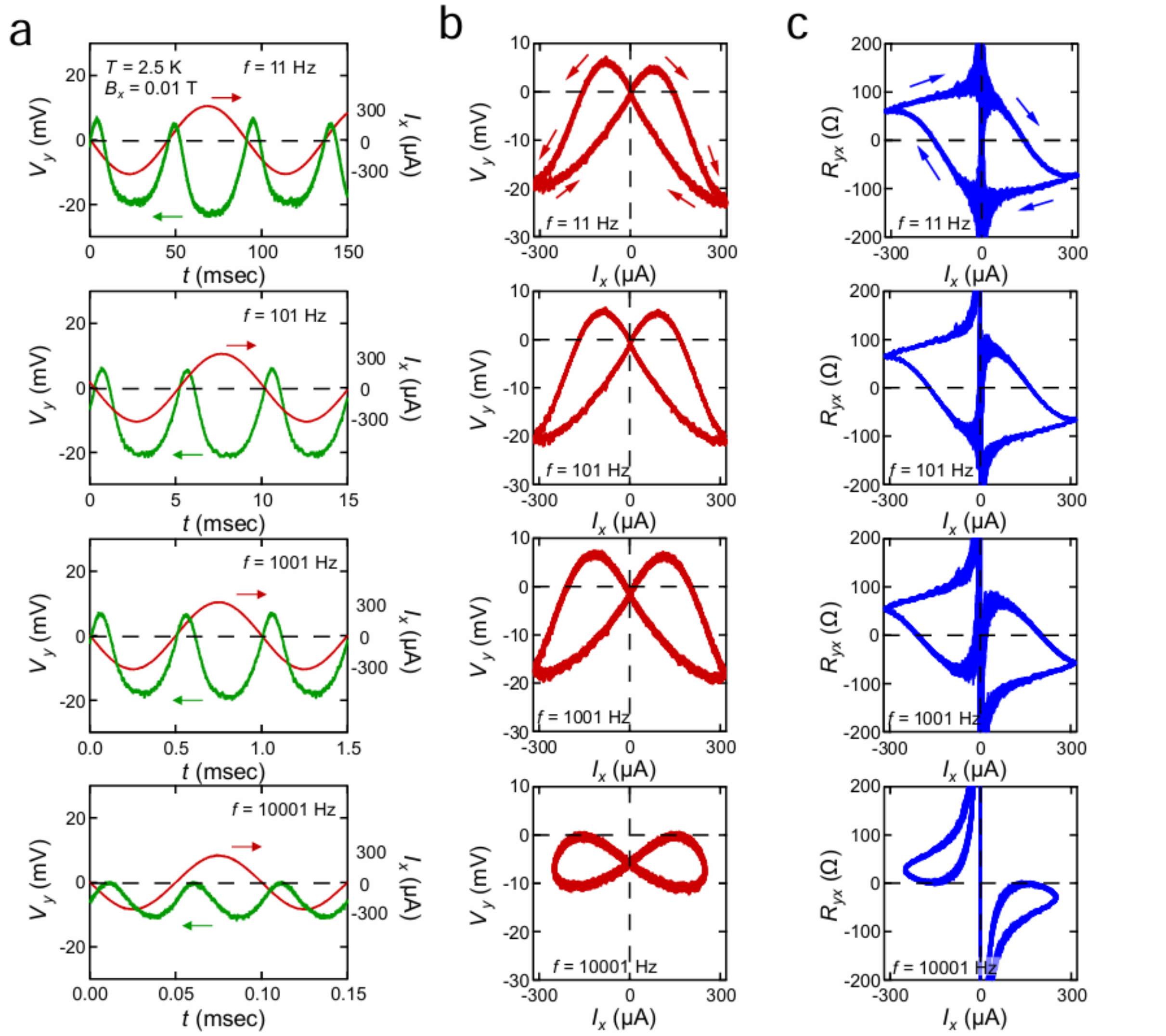

Figure S5: Frequency dependence of AC-induced magnetization reversal. a Hall voltage response (green) and input current (red) with an amplitude of $300\ \mu\text{A}$ and at frequencies of $11, 101, 1001, 10001$ Hz. As the frequency increases beyond 1001 Hz, the amplitude of the measured current decreases possibly due to parasitic capacitance within the electric circuit. **b** Hall voltage vs current characteristics for each frequency. For higher frequencies 1001 Hz and 10001 Hz, the value of $V_y \neq 0$ when $I_x = 0$ because of the trivial phase rotation due to the parasitic capacitance. **c** Hall resistance, calculated from the Hall voltage and current, depending on the current. For all the frequencies, R_{yx} diverges around $I_x = 0$ because of $V_y/I_x = 0/0$.

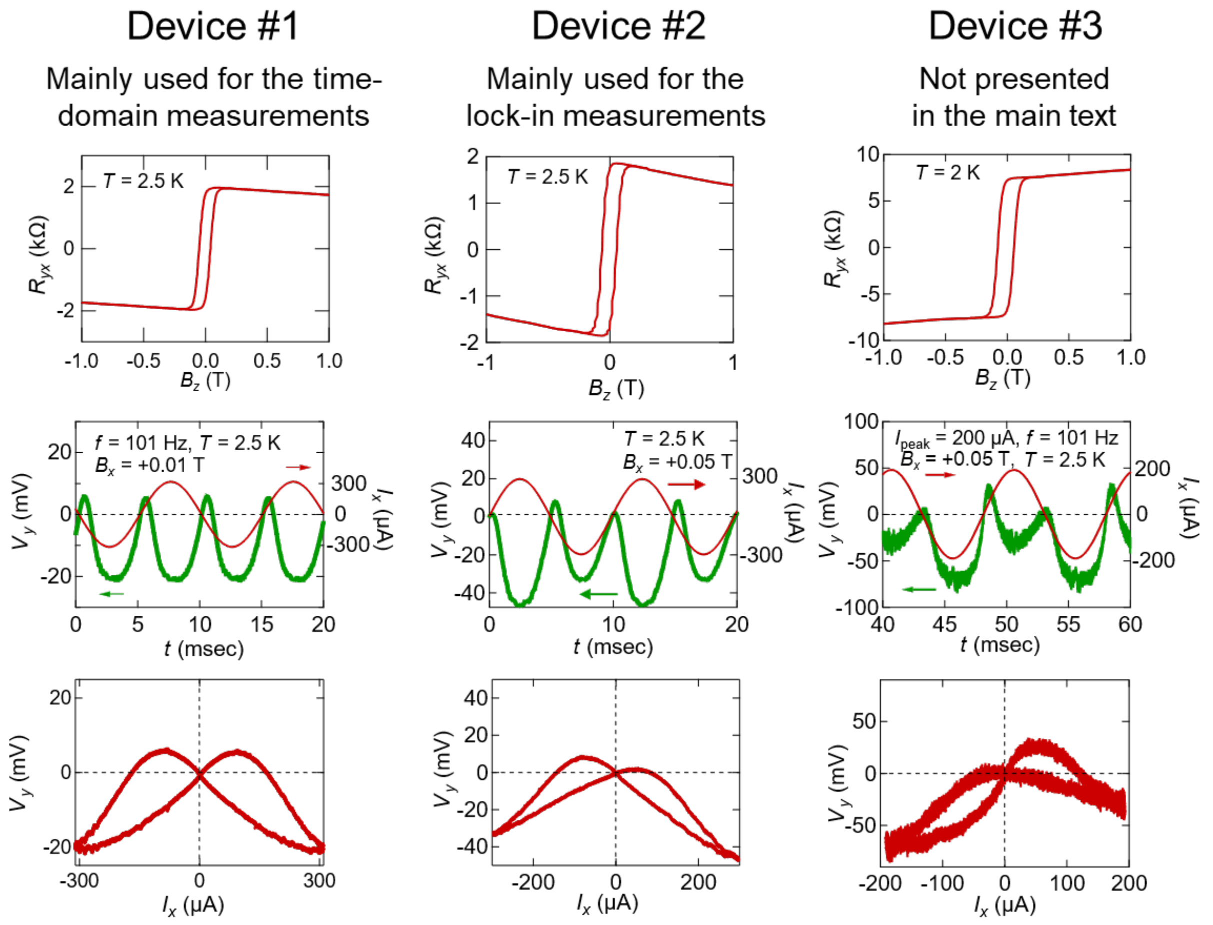

Figure S6: Hall resistivity and basic data in the time-domain measurement for different devices used in our work. Magnetic field (B_x) dependence of Hall resistivity R_{yx} (top) and the waveform of Hall voltage under AC current with a high ampltidue (middle) and the current-Hall voltage characteristics (bottom). The resistivity is measured by DC current of 0.1 μA. The amplitude of the AC current is 300 μA (Device #1, 2) and 300 μA (Device #3), respectively.

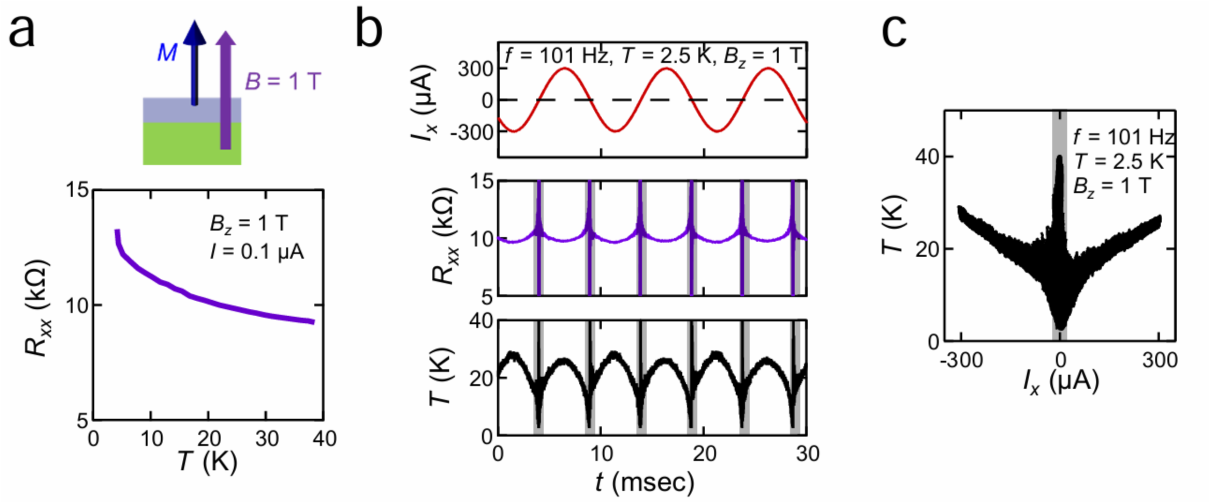

Figure S7: Estimation of temperature increase caused by Joule heating. a Lower part shows the temperature dependence of R_{xx} measured by the probe current as low as $I = 0.1$ μA under the out-of-plane magnetization $B_z = 1$ T (the configuration is shown in the upper part). **b** Time-variation of the current I_x (red), resistance R_{xx} (purple), and temperature T (black) which is estimated from resistance. T is estimated to be as low as 30 K at the highest. R_{xx} and thus T diverge at $I_x = 0$ (the gray areas). **c** Dependence of T on I_x obtained from the $I_x(t)$ and $T(t)$ shown in **b**. T increases as the absolute value of I_x increases. T diverges around $I_x = 0$.

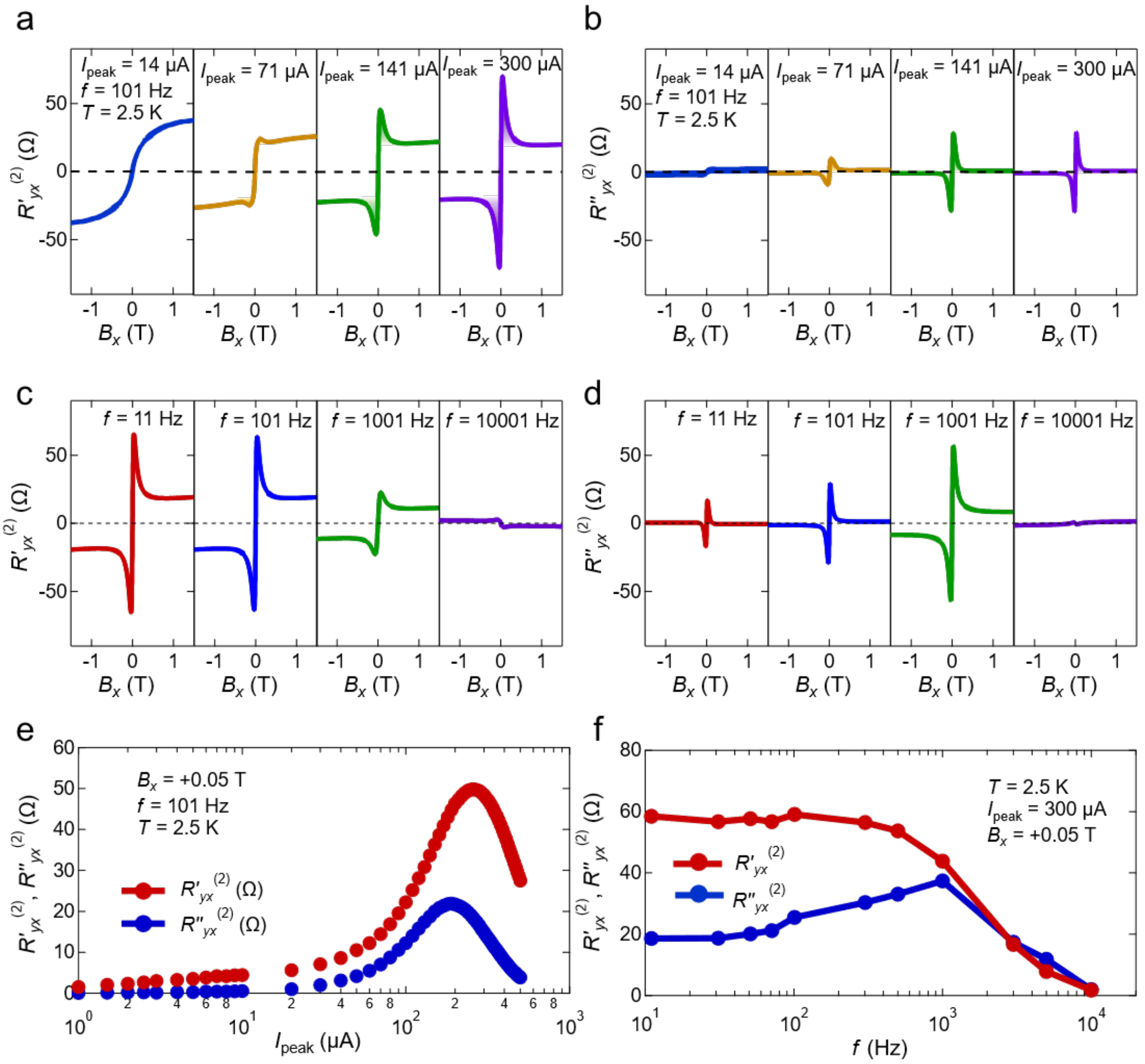

Figure S8: Current- and Frequency-dependence of the second-harmonic Hall responses measured by lock-in amplifier. a, b Magnetic field dependence of $R'^{(2)}_{yx}$ (**a**) and $R''^{(2)}_{yx}$ (**b**), which are defined by $R'^{(2)}_{yx} = V'^{(2)}_{y}/I_{\text{peak}}$ and $R''^{(2)}_{yx} = V''^{(2)}_{y}/I_{\text{peak}}$, respectively. The current amplitudes are 14 μA (blue), 71 μA (yellow), 141 μA (green), 300 μA (purple). **c, d** Magnetic field dependence of $R'^{(2)}_{yx}$ (**c**) and $R''^{(2)}_{yx}$ (**d**) for frequencies 11 Hz (red), 101 Hz (blue), 1001 Hz (green), 10001 Hz (purple). **e, f** Current- (e) and frequency- (f) dependence of second-harmonic Hall responses $R'^{(2)}_{yx}$ (red) and $R''^{(2)}_{yx}$ (blue).

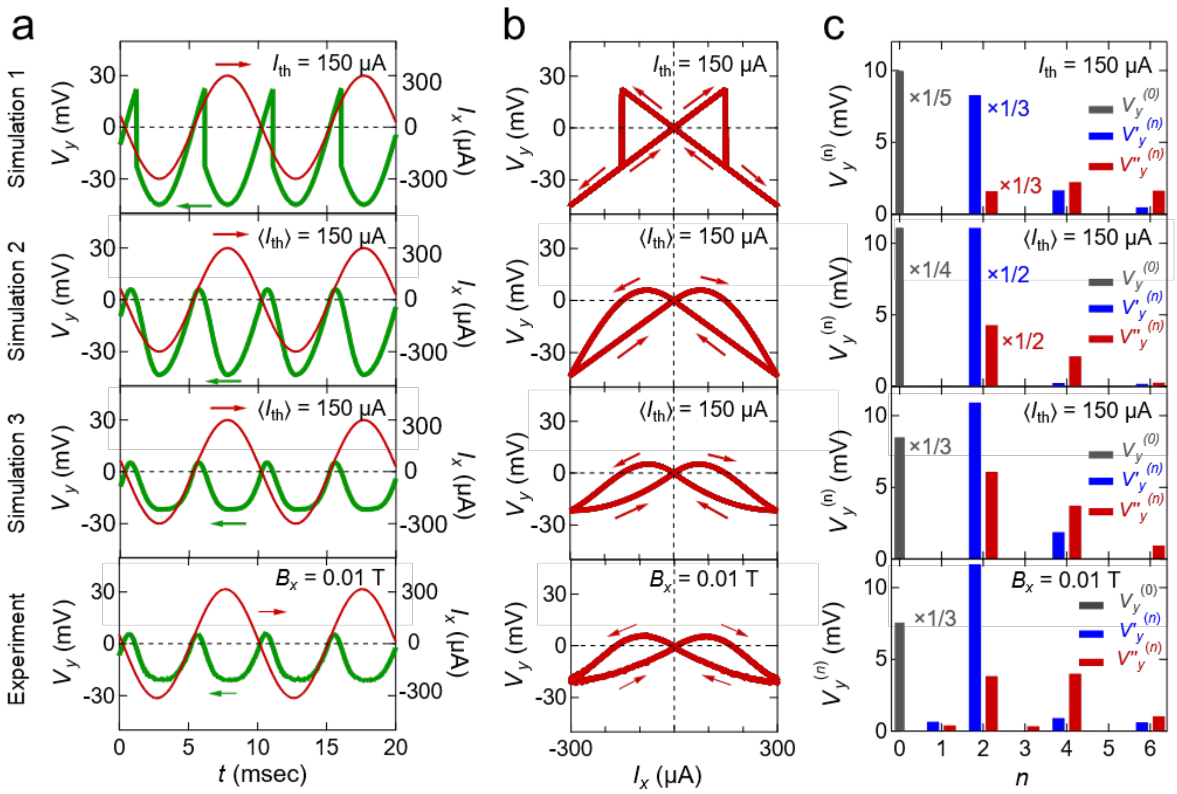

Figure S9: Simple model calculation of magnetization dynamics with and without considering Joule heating and inhomogeneity of magnetic domain. a Waveform of Hall voltage (green) and current (red) for Simulation 1, Simulation 2, Simulation 3, and Experiment. Details of each simulation model are provided in Supplementary Note 6. **b** Hall voltage vs current for Simulation 1, Simulation 2, Simulation 3, and Experiment. **c** Fourier components of the Hall voltage for Simulation 1, Simulation 2, Simulation 3, and Experiment. The blue, red, and gray bars denote the in-phase, out-of-phase, and constant components $V'^{(n)}_y$, $V''^{(n)}_y$, and $V_y^{(0)}$, respectively.

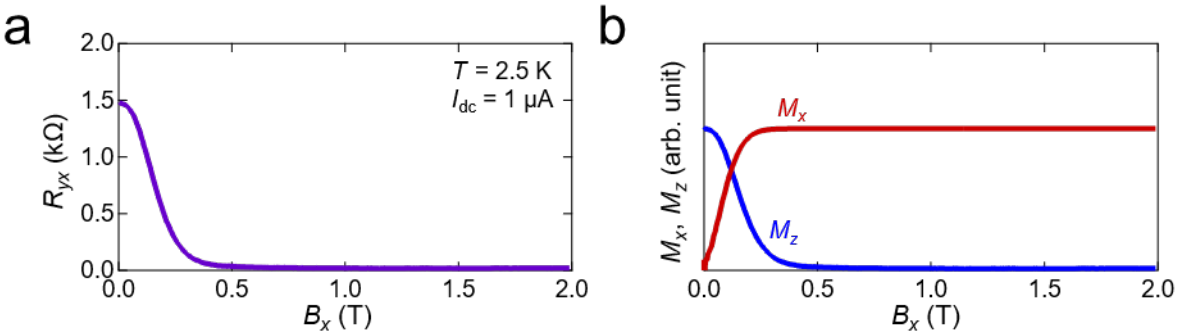

Figure S10: In-plane magnetic field dependence of magnetization direction. a In-plane magnetic field (B_x) dependence of anomalous Hall resistance (R_{yx}) measured by DC current when increasing B_x from 0 T to 2 T after initializing magnetization by out-of-plane magnetic field $B_z = 2$ T . **b** In-plane and out-of-plane components of magnetization M_x (blue) and M_z (red), which are estimated as $M_x \propto \sqrt{(R_{yx}(B_x = 0))^2 - (R_{yx}(B_x))^2}$ and $M_z \propto R_{yx}(B_x)$, respectively, using the values $R_{yx}(B_x)$ shown in **a**.
